# Supplementary material for: Photo-activated raster scanning thermal imaging at sub-diffraction resolution
Source: Nat Commun. 2019 Dec 4;10:5523. doi: 10.1038/s41467-019-13447-0 (PMC6892803; doi:10.1038/s41467-019-13447-0)
Supplement: Supplementary file 1 — Supplementary Information [file 41467_2019_13447_MOESM1_ESM.pdf]

Supplementary Material

Photo-Activated Raster Scanning Thermal Imaging at Sub-Diffraction Resolution

M. Bouzin et al.

Supplementary Note 1: Temperature space and time dependence under modulated laser illumination

The so-called photo-thermal effect occurs when a focused laser beam, with typical visible to near infrared wavelength, primes local light absorption and a consequent heat release in the sample. The laser-induced temperature variation $\Delta T(\mathbf{x},t)$ depends on the intensity profile $I(\mathbf{x},t)$ of the excitation laser beam, on the sample absorption cross-section σ_{exc} at the excitation wavelength and on the thermal properties of the material. The sample thermal diffusivity $D = k/(\rho c)$ is defined in terms of the density ρ, the thermal conductivity k and the specific heat capacity c, and affects both the magnitude and space-time dependence of the temperature variation. $\Delta T(\mathbf{x},t)$ also depends on thermal losses, containing the contribution of thermal radiation under the assumption of grey body emission as well as possible convective heat exchange with the surrounding medium.

We consider at first an ideally thin (2D) opaque and homogeneous sample. The laser-induced temperature variation $\Delta T(x,y,t) = \Delta T(\mathbf{x},t)$ obeys a formal heat transfer equation of the form

$$\frac{\partial \Delta T(\mathbf{x},t)}{\partial t} = D\nabla^2 \Delta T(\mathbf{x},t) + S(\mathbf{x},t) - \frac{1}{\tau_{\mathrm{L}}}\Delta T(\mathbf{x},t) \quad (1)$$

$S(\mathbf{x},t)$ is the volumetric source term accounting for the light absorption process; it is related to the intensity $I(\mathbf{x},t)$ of the excitation beam, to the sample absorption cross-section σ_{exc} and to the number N of absorbers that are hit by the laser beam according to $S(\mathbf{x},t) = N\,\chi\sigma_{\mathrm{exc}} I(\mathbf{x},t)/(V\rho c)$. χ is a constant accounting for the heat release efficiency of the material. τ_{L} is a characteristic losses time: the term $\Delta T(\mathbf{x},t)/\tau_{\mathrm{L}}$ describes both heat conduction toward the sample embedding medium (here, air) and the emission of thermal radiation in the presence of temperature variations limited to a few degrees (for small temperature increments, the Stefan-Boltzmann dependence on the difference of the ambient and sample temperatures elevated at the fourth power can be approximated to a linear dependence on the temperature variation, so that thermal radiation is customarily described by a linear heat transfer term analogous to the one for convection).[1]

The heat equation is conveniently solved in the Fourier k-space. The Fourier transform of Supplementary Equation 1 yields

$$\frac{\partial \widehat{\Delta T}(\mathbf{q},t)}{\partial t} = -D|\mathbf{q}|^2 \widehat{\Delta T}(\mathbf{q},t) + \hat{S}(\mathbf{q},t) - \frac{1}{\tau_{\mathrm{L}}}\widehat{\Delta T}(\mathbf{q},t) \quad (2)$$

$$\rightarrow \quad \frac{\partial \widehat{\Delta T}(\mathbf{q},t)}{\partial t} + \left(D|\mathbf{q}|^2 + \frac{1}{\tau_{\mathrm{L}}}\right)\widehat{\Delta T}(\mathbf{q},t) = \hat{S}(\mathbf{q},t) \quad (3)$$

By introducing

$$\widehat{\Delta \mathbb{T}}(\mathbf{q},t) = \widehat{\Delta T}(\mathbf{q},t) exp\left[\left(D|\mathbf{q}|^2 + \frac{1}{\tau_{\mathrm{L}}}\right)t\right] \quad (4)$$

and by deriving $\widehat{\Delta \mathbb{T}}(\mathbf{q},t)$ in time, we obtain

$$\frac{\partial \widehat{\Delta \mathbb{T}}(\mathbf{q},t)}{\partial t} = \left[\frac{\partial \widehat{\Delta T}(\mathbf{q},t)}{\partial t} + \left(D|\mathbf{q}|^2 + \frac{1}{\tau_{\mathrm{L}}}\right)\widehat{\Delta T}(\mathbf{q},t)\right] exp\left[\left(D|\mathbf{q}|^2 + \frac{1}{\tau_{\mathrm{L}}}\right)t\right]$$

$$= \hat{S}(\mathbf{q},t) exp\left[\left(D|\mathbf{q}|^2 + \frac{1}{\tau_{\mathrm{L}}}\right)t\right] \quad (5)$$

The differential equation (Supplementary Equation 5) leads to

$$\widehat{\Delta \mathbb{T}}(\mathbf{q},t) = \int_0^t \hat{S}(\mathbf{q},t') exp\left[\left(D|\mathbf{q}|^2 + \frac{1}{\tau_{\mathrm{L}}}\right)t'\right] dt' + \widehat{\Delta \mathbb{T}}(\mathbf{q},0) \quad (6)$$

By recalling Supplementary Equation 4,

$$
\begin{cases}
\widehat{\Delta T}(\mathbf{q},t) = \int_0^t \hat{S}(\mathbf{q},t') exp\left[-\left(D|\mathbf{q}|^2 + \frac{1}{\tau_\mathrm{L}}\right)(t-t')\right] dt' + \widehat{\Delta T}(\mathbf{q},0) exp\left[-\left(D|\mathbf{q}|^2 + \frac{1}{\tau_\mathrm{L}}\right) t\right] \\
\Delta T(\mathbf{x},t) = \frac{1}{(2\pi)} \int_{-\infty}^{+\infty} \widehat{\Delta T}(\mathbf{q},t) exp(-i\mathbf{q}\mathbf{x}) d\mathbf{q}
\end{cases} \quad (7)
$$

Supplementary Equation 7 provides the temperature variations $\widehat{\Delta T}(\mathbf{q},t)$ and $\Delta T(\mathbf{x},t)$ in the reciprocal and direct spaces for a general source term $S(\mathbf{x},t)$, i.e., for an unspecified intensity distribution $I(\mathbf{x},t)$ of the excitation laser beam. We are interested here in the temperature variation primed by a laser Gaussian square-wave pulse, with starting time t=0 and pulse extension in time equal to τ_{on}. For the modulated illumination scheme that we always adopt, where the time interval between two consecutive laser pulses is sufficiently long to ensure complete thermal relaxation, the single-pulse laser illumination allows describing the temperature variation at each illumination event. We denote with ω_0^2 the Gaussian beam variance at the focal plane and with $(x_0, y_0) = (0,0)$ the fixed center coordinates of the laser beam (without loss of generality, we center the laser beam at the origin of the reference coordinate system). The Fourier-transformed source term can be therefore expressed as

$$
\hat{S}(\mathbf{q},t') = S_0 exp\left[-\frac{|\mathbf{q}|^2\omega_0^2}{2}\right] \Theta(t')[1-\Theta(t'-\tau_{\mathrm{on}})] \quad (8)
$$

where

$$
\Theta(t_1 - t_2) = \begin{cases} 1 & if \quad t_1 \geq t_2 \\ 0 & if \quad t_1 < t_2 \end{cases} \quad (9)
$$

All the multiplying constants (including the excitation cross-section σ_{exc} and the excitation peak intensity $I(0,0)$) have been inserted into a source amplitude S_0. With the initial condition $\widehat{\Delta T}(\mathbf{q},0) = 0$, by substituting Supplementary Equations 8,9 into the expression for $\widehat{\Delta T}(\mathbf{q},t)$ in Supplementary Equation 7 and by computing the time integral we obtain

$$
\begin{cases}
\widehat{\Delta T}(\mathbf{q},t) = \frac{S_0\tau_\mathrm{L}}{1 + D|\mathbf{q}|^2\tau_\mathrm{L}} \left\{ e^{-\alpha} - e^{-(\alpha+\beta t)} - \left[e^{-\alpha} - e^{-(\alpha+\beta(t-\tau_{\mathrm{on}}))}\right]\Theta(t-\tau_{\mathrm{on}}) \right\} \\
\alpha = \frac{|\mathbf{q}|^2\omega_0^2}{2} \\
\beta = D|\mathbf{q}|^2 + \frac{1}{\tau_\mathrm{L}}
\end{cases} \quad (10)
$$

A fully analytical expression (Supplementary Equation 10) is therefore derived for the temperature variation $\widehat{\Delta T}(\mathbf{q},t)$ in the reciprocal space under a single-pulse laser illumination of a light-absorbing and heat-releasing thin opaque sample. Analytical integration of the temperature profile $\Delta T(\mathbf{x},t)$ in the direct space (Supplementary Equation 7) can be performed instead in the limited time interval $t \in [0, \tau_{\mathrm{on}})$, due to the discontinuity in the primitive function[2]. Inspection of the spatial and temporal dependence of $\Delta T(\mathbf{x},t)$ along the entire time axis requires the numerical integration of Supplementary Equations 7 and 10.

By exploiting the numerical integration of $\widehat{\Delta T}(\mathbf{q},t)$ over $\mathbf{q}$ (Supplementary Equation 10), we have simulated the temporal and spatial profiles of $\Delta T(\mathbf{x},t)$ as a function of the material thermal diffusivity D and of the losses time τ_L. Results are reported in **Supplementary Fig.1**. At each time point t, the temperature increase $\Delta T(\mathbf{x},t)$ primed by a laser square pulse with fixed coordinates (x_0, y_0) is a peaked function in the two-dimensional xy-plane (as exemplified for $t = \tau_{\mathrm{on}}$ in **Supplementary Fig.1a**) and can be approximated by a Gaussian curve of the form

$$
\Delta T(\mathbf{x},t) \cong \Delta T(t) exp\left[-\frac{(\mathbf{x}-\mathbf{x}_\mathrm{c})^2}{2\zeta(t)^2}\right] \quad (11)
$$

ΔT and ζ are the time varying Gaussian amplitude and standard deviation respectively, while $(x_c, y_c) = \mathbf{x}_c$ represent the temperature peak coordinates; they do not necessarily coincide with the beam position (x_0, y_0) but, as described in the main text, they provide the center of the distribution of the light-absorbing entities within the laser spot size.
Beside a linear dependence on the excitation laser intensity, the amplitude $\Delta T(t)$ depends on the material thermal properties, with inverse proportionality to the thermal diffusivity D irrespectively of the losses time τ_L (**Supplementary Fig.1a,b**). $\Delta T(t)$ exhibits an approximately exponential rise and decay as a function of time, similarly to the capacitor voltage of an electrical RC circuit[1] (**Supplementary Fig.1a**):

$$\Delta T(t) \propto \begin{cases} 1 - e^{-\frac{t}{\tau_{\text{rise}}}} & if\ t \leq \tau_{\text{on}} \\ e^{-\frac{t}{\tau_{\text{decay}}}} & if\ t > \tau_{\text{on}} \end{cases} \quad (12)$$

The rise time τ_{rise} only slightly depends on the material thermal properties through D (**Supplementary Fig. 1a**) and is identical irrespectively of the laser activation time and of the laser intensity. It depends on τ_L instead (**Supplementary Fig.1c**). For fixed material, the relaxation time τ_{decay} approaches τ_{rise} for $\tau_{\text{rise}} \ll \tau_{\text{on}}$, whereas it shortens at shorter τ_{on} if the temperature plateau is not reached during the laser activation time. The maximum amplitude $\Delta T(\tau_{\text{on}}) \cong \Delta T(\mathbf{x}_c, \tau_{\text{on}})$ is reached at time τ_{on} and will be hereafter referred to as ΔT_{max}.
The Gaussian standard deviation ζ does not depend on the excitation laser intensity during both laser activation ($t \leq \tau_{\text{on}}$) and thermal relaxation ($t > \tau_{\text{on}}$), but is affected by the losses time τ_L and the material thermal diffusivity (**Supplementary Fig.1d,e**). The higher is D, the larger is the temperature spatial profile at fixed time point t due to the increased heat transfer capability of the material.

The results we have obtained apply to the simple case of a thin, opaque and homogeneous material. More complex cases – including thick materials, with possible semi-transparency in both the visible and far-infrared spectral regions – have already been treated in the literature in the context of the rear-surface flash method for thermal diffusivity measurements.[3-5] These treatments confirm the Gaussian shape of the spatial profiles for laser-primed temperature increments irrespectively of the sample thickness and transparency.[3]
We finally confirm this conclusion by complementing the formal solution of the heat equation (Supplementary Equations 1-10) with the analysis of exemplary experimental data. We investigate the temperature increases detected by a thermal camera under single-pulse laser illumination of synthetic samples with different thermal properties (uniform black ink squares, produced by both microfiche and inkjet printing) analogous to those exploited for the proof-of-principle experiments reported in **Fig.2**. Results, reported in **Supplementary Fig.2**, further demonstrate that a two-dimensional Gaussian function properly approximates the temperature variations primed by the sample absorption of a focused Gaussian laser pulse and imaged by a conventional thermal camera. Therefore, a non-linear surface fit of (regions of interest in) the detected thermal camera frames allows accurate determination of the peaks amplitude and center coordinates, which are the only parameters needed for image reconstruction by photo-activated super-resolution thermal imaging. This conclusion will be reinforced with **Supplementary Fig.4,5**, where we demonstrate that a symmetric Gaussian trial function outperforms asymmetric and skewed fit surfaces in the presence of both homogeneous samples and heterogeneous distributions of absorptive entities inside the excitation laser spot size.

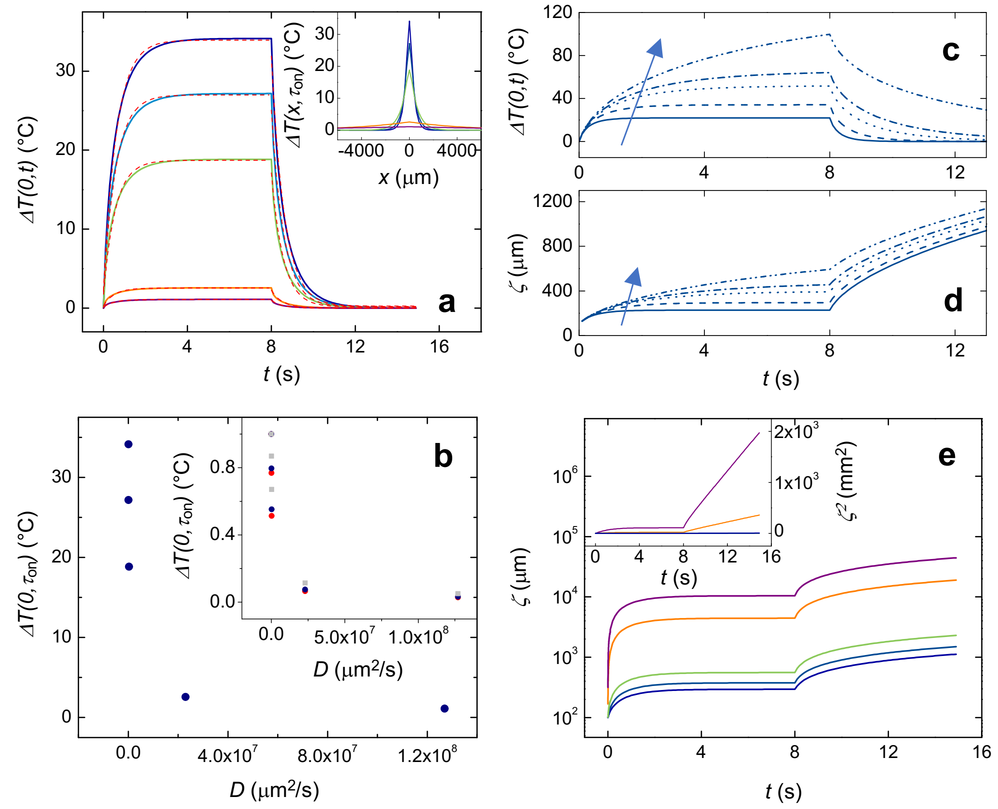

Supplementary Figure 1: theoretical temperature variations primed by single-pulse laser illumination. (a) Simulated temporal profile of the amplitude $\Delta T(x = x_c = 0, t)$ of the temperature variation induced by a laser pulse with duration τ_{on}=8 s impinging on a material with thermal diffusivity D=8·10^4 μm^2/s (PVC, PolyVinyl Cloride, navy), D=1.4·10^5 μm^2/s (water, light blue), D=3.4·10^5 μm^2/s (glass, green), D=2.3·10^7 μm^2/s (bulk iron, orange), D=1.3·10^8 μm^2/s (bulk gold, red). τ_L=1 s, ω_0=100 μm for all the curves. Best fits to Supplementary Equation 12 (dashed red lines) provide τ_{rise}=τ_{decay}=0.62±0.01 s (PVC), 0.59±0.01 s (water), 0.57±0.01 s (glass), 0.5±0.1 s (iron) and 0.5±0.1 s (gold). Inset: spatial profile at time t=τ_{on}=8 s for the same materials of the main panel (identical color code and simulation parameters). (b) Peak amplitude ΔT_{max}=$\Delta T(0, \tau_{on})$ as function of the material thermal diffusivity at fixed source amplitude, τ_L=1 s, ω_0=100 μm, τ_{on}=8 s. Inset: normalized trend of the peak amplitude $\Delta T(0, \tau_{on})$ versus the sample thermal diffusivity at varying losses time (τ_L=10 s red, τ_L=1 s navy, τ_L=0.1 s grey). (c) Effect of τ_L on the amplitude $\Delta T(0, t)$ with τ_{on}=8 s, D=8·10^4 μm^2/s (PVC), ω_0=100 μm; τ_L=0.5, 1, 2, 3, 10 s increasing in the direction of the arrow. (d) Effect of τ_L on the Gaussian standard deviation ζ(t) obtained by the Gaussian fit to Supplementary Equation 11 of the temperature spatial profiles simulated at fixed τ_{on}=8 s, D=8·10^4 μm^2/s (PVC), ω_0=100 μm; τ_L=0.5, 1, 2, 3, 10 s increasing in the direction of the arrow. (e) Time profile of the standard deviation $\zeta(t)$ obtained by the Gaussian fit to Supplementary Equation 11 of the temperature spatial profiles simulated at increasing D values for τ_{on}=8 s, τ_L=1 s, ω_0=100 μm (same D values and color code of panel (a)). A log$_{10}$ scale is adopted on the ζ axis for the sake of display. Inset: ζ^2 temporal profile revealing an expected linear time dependence of the Gaussian variance during thermal relaxation ($t > \tau_{on}$). In both panels (d) and (e) the intercept at t=0 of all the $\zeta(t)$ profiles is equal to ω_0. All the simulations have been performed by the numerical integration of Supplementary Equation 10.

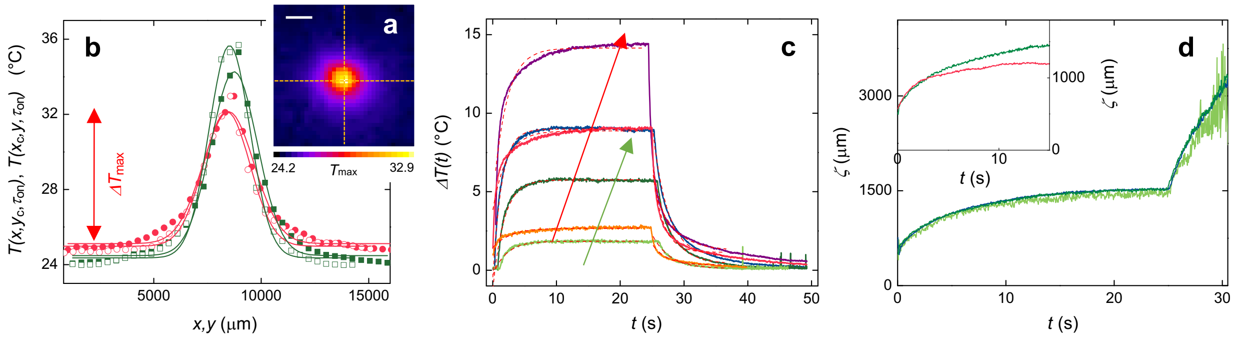

Supplementary Figure 2: experimental temperature variations primed by single-pulse laser illumination. (a) Thermo-camera frame acquired at time $t = \tau_{\rm on}$ by detecting the thermal radiation of a uniform ink square produced by microfiche printing illuminated at its center by a square-wave pulse at 633 nm with $\tau_{\rm on}$=25 s and power P=6 mW. Scale bar = 2850 μm. (b) Red circles: experimental x- and y- temperature profiles indicated by yellow lines in (a) (filled and open symbols, respectively). Profiles are well approximated by the best-fit Gaussian trial function (continuous lines, Supplementary Equation 11) providing the amplitude $\Delta T_{\rm max}$ and peak coordinates $(x_{\rm c}, y_{\rm c})$. Green curves: x- and y- temperature profiles (filled and open symbols, respectively) extracted from the camera frame at time t=$\tau_{\rm on}$=25 s resulting from laser-pulse illumination of a black ink square produced by inkjet printing on white paper. Continuous lines are the best Gaussian fit to Supplementary Equation 11. (c) Time dependence of the amplitude ΔT obtained by the 2D Gaussian fit to Supplementary Equation 11 of the temperature variations induced by a single pulse with duration $\tau_{\rm on}$=25 s impinging at the center of uniform black ink squares produced by microfiche and inkjet printing (green and red shades, respectively). Excitation power P=3 mW, 6 mW and 9 mW, increasing in the direction of the arrows. Dashed red lines are the best fits to Supplementary Equation 12. (d) Time profile of the standard deviation ζ(t) obtained by the surface Gaussian fit to Supplementary Equation 11 of the experimental temperature peaks imaged by the thermal camera and induced at the center of the uniform microfiche ink square with a square-wave laser pulse ($\tau_{\rm on}$=25 s); laser power on the sample plane P=9 mW (navy), 6 mW (green) and 3 mW (light green). Twice the intercept $2\zeta(0)$=(1200±180) μm provides an estimate for the resolution of the thermal camera in its conventional operation. Inset: comparison of the standard deviation time profiles recovered under identical conditions (P=6 mW) on the microfiche and inkjet-printed samples (green and red, respectively). The intercept $2\zeta(0)$ is the same for both materials. Excitation beam 1/e^2 diameter = (56±2) μm for all the curves.

Supplementary Note 2: Choice of Δx and Δt parameters

Results described in **Supplementary Note 1** and reported in **Supplementary Fig.1,2** provide the criteria in the selection of the minimum distance **Δx** and the minimum time interval Δt between a pair of consecutive illumination events in the modulated illumination scheme described in the main text and in **Supplementary Fig.3b**. The goal is to guarantee that two consecutive laser-induced temperature variations appear as (spatially and/or temporally) separate peaks in the thermal camera images. Based on **Supplementary Fig.1,2**, for a laser pulse centered at (x_0, y_0) starting at t=0 with duration $\tau_{\rm on}$ the temperature difference drops at 1/e^3 ≅ 5% of the peak value after a time interval $\tau_{\rm on} + 3\tau_{\rm decay}$ (Supplementary Equation 12), and after such a time interval the spatial width (1/e^2 radius) of the detected temperature profile equals $2\zeta(\tau_{\rm on} + 3\tau_{\rm decay})$ (Supplementary Equation 11). In other words, a minimum time interval $\Delta t = \tau_{\rm on} + 3\tau_{\rm decay}$ has to lapse before a point within a radius $2\zeta(\tau_{\rm on} + 3\tau_{\rm decay})$ from (x_0, y_0) gets illuminated again. Any pair $(\Delta\mathbf{x}, \Delta t)$ satisfying this constraint can in principle be selected; we report details on the values we have adopted for $(\Delta\mathbf{x}, \Delta t)$ in the captions of **Fig.2,3**. We remark that ζ and $\tau_{\rm decay}$ implicitly contain the dependence on the sample thermal diffusivity according to **Supplementary Note 1**. When the thermal properties of the investigated material are not known a priori, $\tau_{\rm decay}$ and $2\zeta(\tau_{\rm on} + 3\tau_{\rm decay})$ can be easily quantified experimentally by illuminating the sample with even a single laser pulse lasting $\tau_{\rm on}$, and by measuring the decay time of the $\Delta T(\mathbf{x}_{\rm c}, t)$-vs-time plot and the width of the $\Delta T(\mathbf{x}, \tau_{\rm on} + 3\tau_{\rm decay})$-vs-$\mathbf{x}$ plot provided by the thermo-camera image stack.

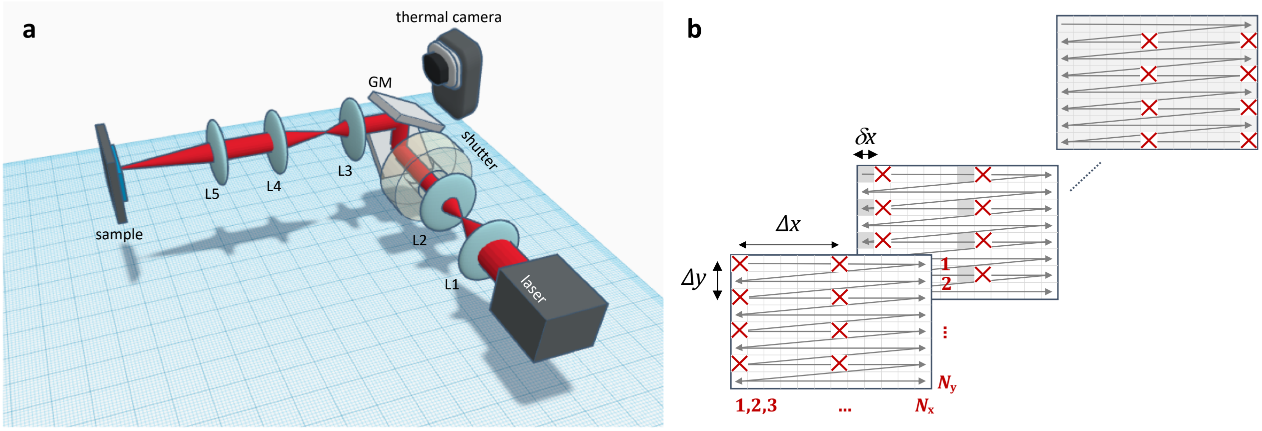

Supplementary Figure 3: photo-thermal imaging setup. (a) Schematic of the setup employed for photo-activated thermal imaging at sub-diffraction resolution, as described in the *Methods* section of the main text (L1-L5, lenses; GM, galvanometric mirrors). (b) Schematic of the adopted raster-scanning modulated illumination scheme, depicted here with the first, second and last scans (from left to right) of a hypothetical exemplary acquisition. The laser beam is scanned uni-directionally (grey arrows) along a conventional raster path with grid size N_xxN_y and pixel size δx, but during a single complete raster scan illumination is only allowed (i.e., the shutter is only opened) on a limited set of isolated pixels (red crosses; grey pixels in each panel are those that have been illuminated during previous scans, whereas white pixels are those still to be illuminated by the laser beam). Δx is the minimum distance (in pixel units) between two consecutively illuminated pixels along the *x*-direction, while Δy defines the distance between the nearest lines where the shutter can be opened (for convenience sake, values for Δx and Δy are chosen among the divisors of N_x and N_y, respectively). The raster scan has to be repeated $\Delta x \Delta y$ times, with shifted sets of illuminated pixels, in order to illuminate the entire grid at least once. With laser dwell times τ_{on} and τ_{off} during laser activation and de-activation, the time required to scan the raster path once is $t_{scan} = N_x N_y \tau_{on}/(\Delta x \Delta y) + N_x N_y \tau_{off}[1 - 1/(\Delta x \Delta y)]$. The total data acquisition time is therefore $t_{tot} = t_{scan} \Delta x \Delta y$.

Supplementary Note 3: Resolution of conventional diffraction-limited thermal imaging

The spatial resolution Δr of conventional thermal imaging can be evaluated by quantifying the width of the 2D spots produced on the thermo-camera images by sub-resolved objects heated to some constant temperature T above the room temperature T_0. If we denote with $\zeta(t)$ the standard deviation of the temperature profile $\Delta T(\mathbf{x}, t)$ induced at time point t by a Gaussian laser pulse, we could theoretically obtain an estimate for Δr from the intercept at t=0 of the $2\zeta(t)$ temporal profile. Based on the solution of the heat equation, we expect the intercept to theoretically coincide with twice the standard deviation ω_0 of the Gaussian excitation laser spot on the focal plane, irrespectively of the thermal properties of the material (**Supplementary Fig.1d,e**). However in practice, since the temperature variation is monitored by the thermal camera based on the intensity of the infrared radiation emitted by the sample, a practical limit to the attainable spatial resolution is set by diffraction at the thermal camera collecting lens. According to Abbe's law,

$$\Delta r \geq 0.61 \frac{\lambda}{\mathrm{N.A.}} \qquad (13)$$

where λ is the detected light wavelength and N.A. is the system numerical aperture. For commercially available low-cost thermal cameras the numerical aperture is typically low (N.A. = 0.023 in the present case, where a lens with focal lens f=1.8 cm and f-number $f_\#$=1.3 operates at a distance $d \cong 30$ cm from the sample plane). At the longest detected wavelength λ = 13 μm, N.A. = 0.023 results in a best attainable resolution Δr = 345 μm (> $2\omega_0$~20-50 μm for all the experiments of the present work). Other factors influencing the effective value for Δr include the extended pixel size of the thermal camera on the sample plane (here, ~ 400 μm), and the eventual presence of signal cross-talk among the micro-bolometers constituting the thermo-camera sensor. Globally, the effective resolution quantified by the intercept at

t=0 of the $2\zeta(t)$ temporal profile measured experimentally on the setup employed here equals Δr = (1200±180) μm (**Supplementary Fig.2c**). We remark that this estimate does not depend on the laser activation time and intensity, and on the investigated material (**Supplementary Fig.2c**). It is exclusively related to the operation of the adopted thermal camera, and represents the resolution that would be ideally obtained if a sufficiently high temperature variation (i.e., above the thermal camera sensitivity σ_T = 0.1°C) could be measured at the very beginning (t=0) of the sample illumination.
In practice, when temperature variations are externally induced by the sample absorption of laser light, a time t elapses before the observed $\Delta T(\mathbf{x}, t)$ exceeds σ_T. At the time point of image acquisition, heat diffusion from the heated objects to the surroundings already leads to an enlargement of the detected temperature profiles, so that identical-emissivity objects have to be at a distance $\Delta r_{eff}>\Delta r$ to be distinguished. Resolution is lowered therefore depending on the thermal conductivity properties of the medium where emitting objects are embedded. This is exemplified in **Supplementary Fig.7** with a second measurement of the resolution of the thermal camera, performed on the same synthetic samples exploited (**Fig.2**) for the proof-of-principle sub-diffraction imaging experiments. Two sub-resolved parallel ink stripes, 90-μm in width and separated by a 3.3 mm distance, have been printed on a microfiche support (**Supplementary Fig.8** and *Methods*). The ink absorption at 633 nm has been primed during a fast bidirectional raster-scanning across the entire sample, in the absence of laser light modulation, and a thermal image of the stripes has been obtained (**Supplementary Fig.8**) as a signal maximum projection over the image stack simultaneously collected by the thermo-camera. A two-component 1D Gaussian fitting of the induced temperature profile along the direction orthogonal to the stripes provides an average full width at 60% amplitude of (1850±80) μm, that can be interpreted as an effective, sample- and laser-power dependent, resolution of the thermo-camera in its conventional operation under imaging conditions similar to those employed for the experiments reported in **Fig.2**.

Supplementary Note 4: Two-dimensional Gaussian fit of thermographic images

As previously discussed with **Supplementary Note 1** and **Supplementary Fig.1,2**, a 2D symmetric Gaussian function well approximates the temperature increase imaged by the thermal camera and primed by a focused laser beam impinging on an extended uniform absorbing sample. Arguing that the presence of non-uniform distributions of absorbing entities (i.e., heterogeneities in local thermal properties) inside the excitation laser spot might lead to asymmetric temperature profiles in the thermo-camera images, we have optimized the fitting routine by testing different types of bell-shaped surfaces:

(i) The symmetric Gaussian of Supplementary Equation 11, with amplitude ΔT, peak coordinates (x_c, y_c), standard deviation ζ and baseline T_0:

$$\Delta T(x,y,t) = \Delta T exp\left[-\frac{(x-x_c)^2+(y-y_c)^2}{2\zeta^2}\right] + T_0 \quad (14)$$

(ii) An asymmetric 2D Gaussian with different standard deviations ζ_x and ζ_y along the x- and y-directions and orientation defined by the angle θ:

$$\begin{cases} \Delta T(x,y,t) = \Delta T exp[-(a(x-x_c)^2 + 2b(x-x_c)(y-y_c) + c(y-y_c)^2)] + T_0 \\ a = \frac{cos^2\theta}{2\zeta_x^2} + \frac{sin^2\theta}{2\zeta_y^2} \\ b = -\frac{sin2\theta}{4\zeta_x^2} + \frac{sin2\theta}{4\zeta_y^2} \\ c = \frac{sin^2\theta}{2\zeta_x^2} + \frac{cos^2\theta}{2\zeta_y^2} \end{cases} \quad (15)$$

(iii) A Skewed normal distribution[3,4] with skewness parameters α_1 and α_2:

$$\Delta T(x,y,t) = \Delta T exp\left[-\frac{(x-x_c)^2+(y-y_c)^2}{2\zeta^2}\right]\left[1 + erf\left(\alpha_1\frac{x-x_c}{\sqrt{2}\zeta}\right)\right]\left[1 + erf\left(\alpha_2\frac{y-y_c}{\sqrt{2}\zeta}\right)\right] + T_0 \quad (16)$$

A fourth fitting procedure (iv) has been finally tested: a first non-linear fit to the symmetric Gaussian of Supplementary Equation 14 is performed with fixed variance to provide the peak coordinates, which are fixed in a second fit to recover the Gaussian variance and the amplitude.

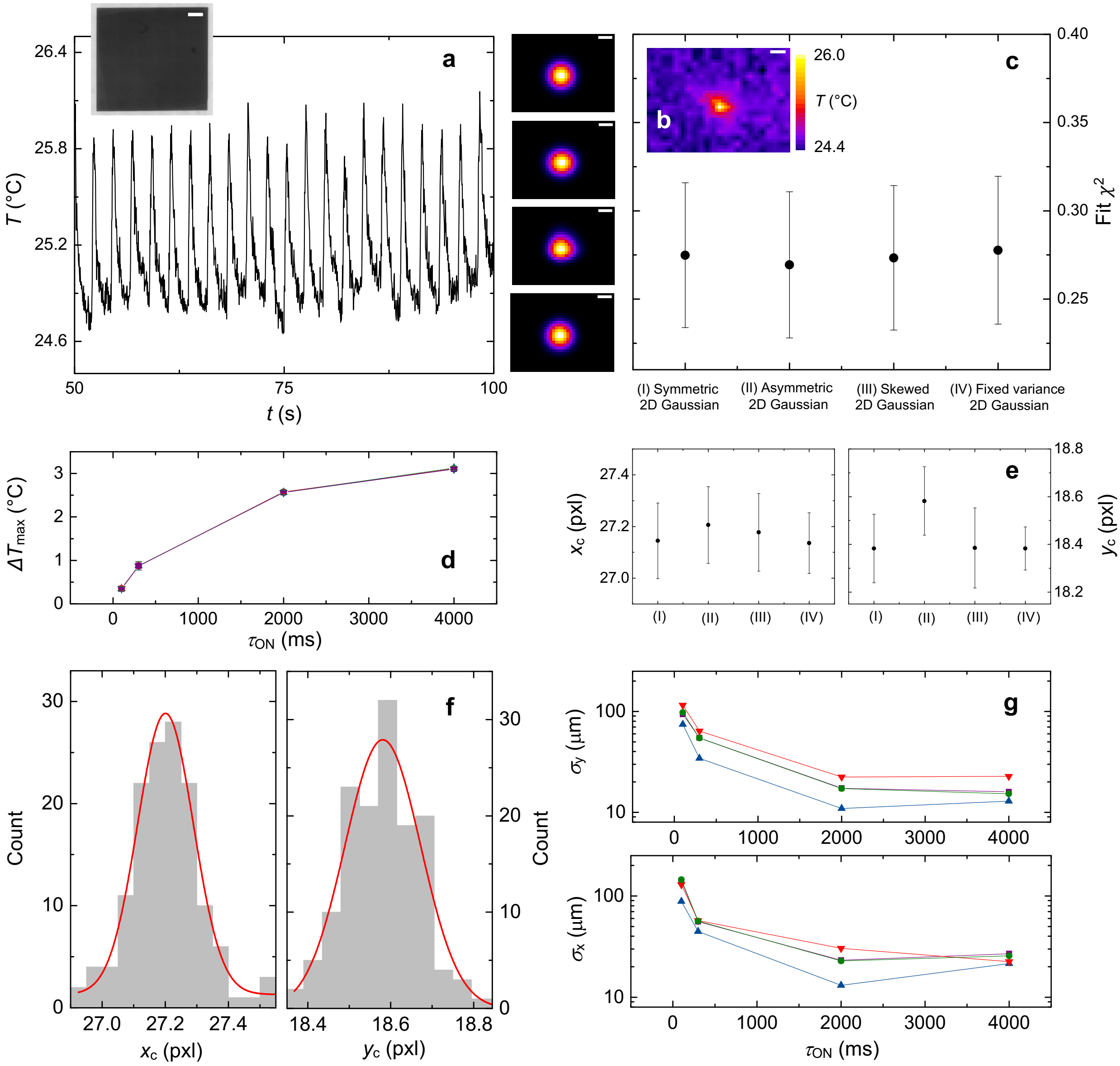

Supplementary Figure 4: localization uncertainty (I). (a) Temperature-vs-time profile resulting from a periodical square-wave illumination (τ_{on}=300 ms, τ_{off}=2s) of a microfiche ink square; at each time point the temperature is reported as the maximum value in a ROI of the thermo-camera image centered on the temperature increase. Inset: transmitted-light image of the sample at 633 nm (500-µm scale bar). (b) Average ROI from five images acquired at the end of a 300-ms illumination event on the sample in (a). Left (top to bottom): surface fits of panel (b) to (i) a symmetric Gaussian, (ii) an asymmetric Gaussian, (iii) a skewed Gaussian and (iv) a symmetric Gaussian with two-step fit (Supplementary Equations 14-16). Scale bars=1.9 mm. (c) χ^2 of the surface fit of the thermo-camera ROI to the four fitting procedures of **Supplementary Note 4**. (d) Best-fit ΔT_{max} versus τ_{on}, provided by the fit of the thermo-camera ROI to a symmetric Gaussian (violet), an asymmetric Gaussian (green), a skewed Gaussian (red) and a symmetric Gaussian with two-step fit (blue). (e) Best-fit coordinates x_c and y_c recovered from the fit of the thermo-camera ROI to the trial functions (i)-(iv) of **Supplementary Note 4**. Data in (c)-(e) are mean±std.dev. over 150 consecutive temperature peaks primed on the sample in (a) (τ_{on}=300 ms in (c) and (e)); for each illumination event, the average of five frames around τ_{on} has been exploited for fitting. (f) Histograms of x_c and y_c (two-step fit) recovered from 150 temperature

peaks induced at τ_{on}=300 ms on the sample in (a), overlaid to the Gaussian fits (red lines). (g) Uncertainties σ_x and σ_y in the localization of the peak coordinates x_c and y_c recovered from the fit of the thermo-camera ROI to a symmetric Gaussian (violet), an asymmetric Gaussian (green), a skewed Gaussian (red) and a symmetric Gaussian with two-step fit (blue). σ_x and σ_y have been quantified as standard deviations of the corresponding histograms for x_c and y_c based on 150 temperature peaks. The two-step fit systematically minimizes σ_x and σ_y. A power P=4.6 mW and an excitation beam 1/e² diameter = 56±2 μm have been exploited for all the measurements.

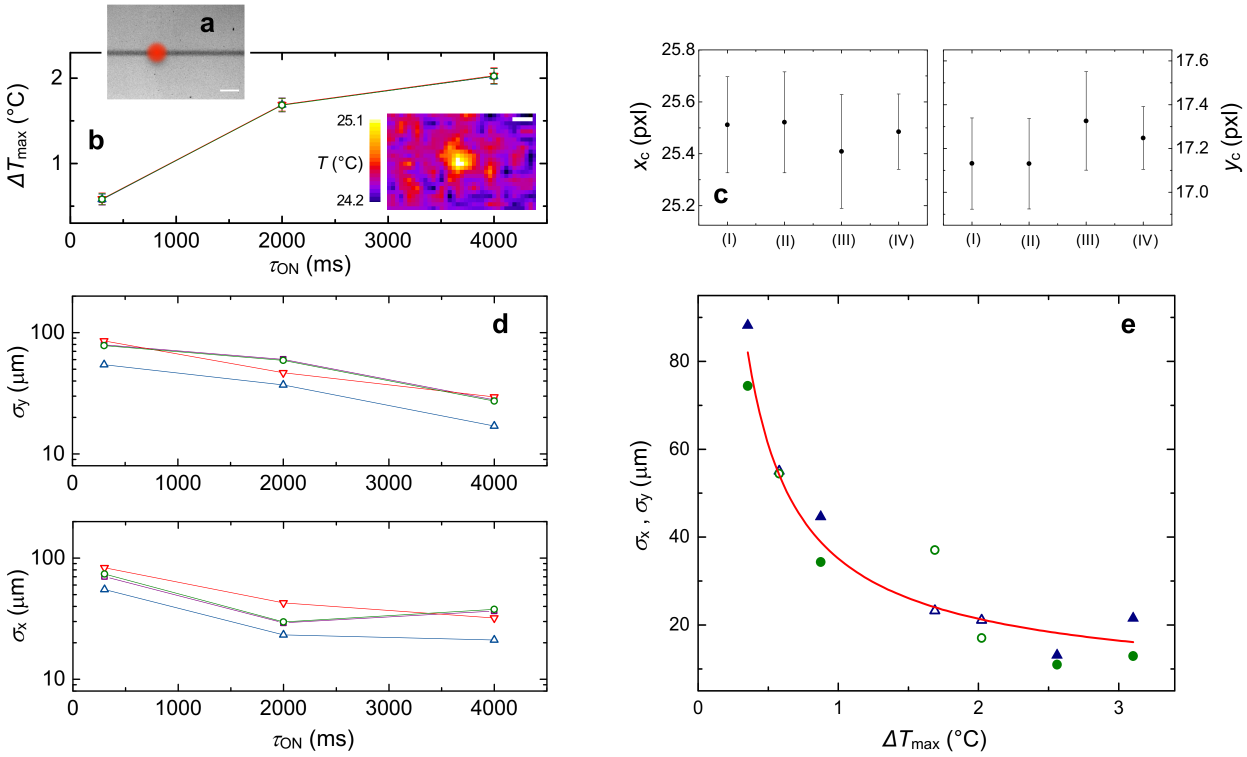

Supplementary Figure 5: localization uncertainty (II). (a) Transmitted-light image at 633 nm of the uniform ink stripe (30-μm width) printed as microfiche and employed for the quantification of the peak-localization uncertainty. The sketch of the excitation beam (red spot) indicates the fixed position of the beam for the sample illumination with a periodical square-wave laser pulse of duration τ_{on}. Scale bar=100 μm. (b) Best-fit amplitude ΔT_{max} (mean±std.dev. over N=100 consecutive temperature peaks) versus τ_{on} on the sample in (a), provided by the fit of the thermo-camera ROI to a symmetric Gaussian (violet), an asymmetric Gaussian (green), a skewed Gaussian (red) and a symmetric Gaussian with two-step fit (blue). For each illumination event, the average of five frames detected around τ_{on} has been exploited for surface fitting. (c) Best-fit peak coordinates x_c and y_c (mean±std.dev., N=100) recovered from the fit to the trial functions (i)-(iv) of **Supplementary Note 4** of the average of five thermo-camera frames collected at the end of 100 consecutive temperature peaks induced on the sample in (a) with τ_{on}=300 ms. (d) Localization uncertainties σ_x and σ_y on the peak coordinates x_c and y_c recovered from the fit of 100 consecutive temperature peaks at increasing τ_{on} on the sample in (a); colors are coding for the surface fit of the thermo-camera frames, performed according to a symmetric Gaussian (violet), an asymmetric Gaussian (green), a skewed Gaussian (red) and a symmetric Gaussian with two-step fit (blue). (e) Localization uncertainties σ_x (triangles) and σ_y (circles) on the peak coordinates x_c and y_c reported as a function of ΔT_{max} for the microfiche ink square (filled symbols) and the microfiche ink stripe (open symbols). The red solid line is the best global fit to $\sigma_{x,y} \approx \sqrt{\alpha/\Delta T_{max} + \beta/\Delta T_{max}^{2}}$ with parameters α=599±221 °Cμm² and β=634±133 °C²μm². A power P=4.6 mW and an excitation beam 1/e² diameter=56±2 μm have been exploited for all the measurements.

Supplementary Note 5

5.1 Thermal camera imaging procedure and viewing angle effect

When observing an opaque grey-body with emissivity ε at temperature T_{obj}, the thermal camera senses a total radiation power[1,8]

$$\Phi_{\mathrm{tot}} = \varepsilon\tau\Phi_{\mathrm{obj}}(T_{\mathrm{obj}}) + (1-\varepsilon)\tau\Phi_{\mathrm{amb}}(T_{\mathrm{amb}}) + (1-\tau)\Phi_{\mathrm{atm}}(T_{\mathrm{atm}}) \qquad (17)$$

where:

- $\varepsilon\tau\Phi_{\mathrm{obj}}(T_{\mathrm{obj}})$ is the radiant power contribution of the grey-body, with $\Phi_{\mathrm{obj}}(T_{\mathrm{obj}})$ corresponding to the radiant power detected by the thermal camera in the presence of a black-body at the object temperature T_{obj}. τ is the atmosphere transmittance and describes the attenuation of the emitted radiation in the atmosphere across the camera-sample distance d.
- $(1-\varepsilon)\tau\Phi_{\mathrm{amb}}(T_{\mathrm{amb}})$ accounts for the atmosphere-attenuated thermal radiation emitted by the surroundings at temperature T_{amb} and reflected by the grey-body with reflectance $(1-\varepsilon)$.
- $(1-\tau)\Phi_{\mathrm{atm}}(T_{\mathrm{atm}})$ is the radiant power contribution of the atmosphere at temperature T_{atm}, with emissivity $(1-\tau)$.

Based on Supplementary Equation 17, the radiant power $\Phi_{\mathrm{obj}}(T_{\mathrm{obj}})$ can be retrieved starting from the measured sensor signal Φ_{tot} as[1,8]

$$\Phi_{\mathrm{obj}}(T_{\mathrm{obj}}) = \frac{1}{\varepsilon\tau}\Phi_{\mathrm{tot}} - \frac{1-\varepsilon}{\varepsilon}\Phi_{\mathrm{amb}}(T_{\mathrm{amb}}) - \frac{1-\tau}{\varepsilon\tau}\Phi_{\mathrm{atm}}(T_{\mathrm{atm}}) \qquad (18)$$

In order for $\Phi_{\mathrm{obj}}(T_{\mathrm{obj}})$ to be evaluated via Supplementary Equation 18, the grey-body emissivity has to be provided to the thermo-camera acquisition software. The atmospheric transmittance τ is derived instead by the same software once the atmospheric temperature T_{atm}, the relative humidity and the camera-sample distance d are provided as input parameters. Together with T_{atm}, the ambient temperature T_{amb} has to be provided to the camera software to enable computation of the two contributions $\Phi_{\mathrm{amb}}(T_{\mathrm{amb}})$ and $\Phi_{\mathrm{atm}}(T_{\mathrm{atm}})$ based on the calibration curve stored in the thermal-camera firmware. Such a calibration provides the relation between detected camera signal and black-body temperature, and is also at the basis of the final conversion of the experimental value $\Phi_{\mathrm{obj}}(T_{\mathrm{obj}})$ into the object temperature T_{obj}.

It is worth remarking that the camera calibration (provided with the camera by the manufacturer) relates the black-body temperature to the actual detected sensor signal. Therefore, all the camera properties (e.g., spectral response of the detector, transmittance of the optics) affecting the sensor signal are automatically accounted for in the calibration curve.

All FLIR imaging systems adopt Supplementary Equation 17 as general measurement formula[8]: provided ε, τ, T_{atm} and T_{amb} are known, it allows deriving temperature values (in the present work, laser-primed temperature variations) based on the number of infrared photons reaching the thermal camera sensor.

Supplementary Equation 17 assumes however the thermal camera is pointing to the sample along the normal direction. Any thermal camera tilt and the resulting viewing angle with respect to the sample might affect (i) the size of imaged objects, distances being subject to the foreshortening effect of perspective view, and (ii) measured temperature values due to the possible angular dependence of the sample emissivity.

Based on these considerations, we investigate in the following the effect of our thermal camera orientation and quantify the emissivity for all the samples employed in the experiments of the present work.

5.1.1 Viewing angle effect on the size of imaged objects

All the experiments of the present work have been performed with the thermal camera facing the laser-illuminated surface of the sample along the normal direction in the *xz*-plane, with the only tilt along the vertical direction never exceeding 20° (**Supplementary Fig.14a**, where the adopted imaging geometry corresponds to ϕ = 0° and θ = 10°-20°).

Even under the imaging configuration corresponding to the highest deviation from normal observation (ϕ = 0°, θ = 20°), the 20° thermal camera tilt along the vertical axis enables accurate imaging of object sizes across large (cm^2) areas of the field of view. Indeed, the thermographic image of a heated object of known size and shape (a glass slide 7.65cm x 2.5cm in size) only reveals a 3% variation of the object width across the 2.5cm distance along the vertical direction (**Supplementary Fig.15a,b**). The object width on the thermal image (varying from 197 to 191 pixels) can be exploited to derive the horizontal thermo-camera pixel size δx_T on the sample plane, and this 3% variation only translates into a 2% uncertainty on the pixel size (δx_T = 395±8 μm). As expected instead from the absence of any thermo-camera tilt in the *xz*-plane, nearly no perspective effect is observed for the imaged height of the glass slide: the 2.5cm imaged object height shows a 1.6% variation across the whole 7.65-cm length, resulting in a 1% uncertainty on the vertical pixel size (δy_T = 404±4 μm) (**Supplementary Fig.15a,c**). Significantly, the absence of relevant perspective effect over ~20 cm^2 areas definitely excludes foreshortening effects over the smaller ~mm^2 areas we have imaged in the experiments of **Fig. 2,3**.
It is finally to be noted that the compatibility between the retrieved δx_T and δy_T pixel sizes along the *x*- and *y*- directions justifies our assumption of a square thermo-camera pixel size on the sample plane, to be exploited for the localization of laser-induced temperature variations and image reconstruction during super-resolution imaging experiments.

5.1.2 Viewing angle effect on measured temperature increments: emissivity measurements

While black-bodies behave like perfect isotropically diffuse emitters, real grey-body surfaces may display a dependence of the emissivity ε on the angle of observation with respect to the surface normal.[1,9] It is therefore important that the emissivity is measured at the very same detection angle subsequently exploited for photo-thermal imaging.
The emissivity of both synthetic and biological samples has been measured following the black-tape method suggested in the literature.[1] Half of the sample is covered by black matte tape of known angle-independent emissivity (ε_{tape} = 0.95) and, under thermal equilibrium, two regions of interest (ROIs) centered on the tape and on the sample, respectively, are selected on the thermal camera frames. While the emissivity ε_{tape} is employed for the temperature measurement in the tape ROI, an emissivity ε is set on the sample: the correct sample emissivity ε can then be found by varying it in the camera software until the thermal image provides the same temperature for the tape and sample ROIs. If the procedure is repeated by rotating the sample with respect to the thermal camera surface normal, the angle-dependence of the emissivity can be accessed and quantified as schematically depicted in **Supplementary Fig.14**.
The emissivity of microfiche samples has been measured on ink-printed areas at increasing tilt of the sample with respect to the thermal camera, covering the broad range θ = 0°-40° (at fixed ϕ = 0°). No noticeable dependence on the angle of observation has been revealed in the experimental emissivity values (**Supplementary Fig.14**) up to a 20° tilt (results agree with the literature, reporting constant emissivity from the normal direction up to at least θ = 40°-45° for the majority of materials[1]). The average value ε = 0.80 recovered under the typical imaging geometry (ϕ = 0° and θ = 0-20°) has been therefore exploited for the analysis of photo-activated super-resolution thermal imaging experiments on microfiche samples.
The emissivity of nanoparticle-treated skin sections has been measured under the configuration (ϕ = 0° and θ = 20°) of the experiments reported in **Fig.3**. We can assume that the sample emissivity is everywhere comprised between: (i) the emissivity ε_{glass} of the bare glass coverslip, and (ii) the emissivity ε_{PBNP} of a solution of PBNPs cast on the glass coverslip at the very same concentration *C* employed for the NPs injection in the treated tissue section. Indeed, ε_{glass} would apply to all the pixels of the reconstructed image located outside the skin section, whereas ε_{PBNP} would apply to the pixels containing the nanoparticles at the highest possible concentration *C*. Since the remaining pixels would contain an intermediate local concentration of nanoparticles, it is reasonable to assume the emissivity in those pixels could not exceed ε_{PBNP}. When the nanoparticles distribution and local concentrations are not known a priori (as in the present case), the range ε_{glass}-ε_{PBNP} provides the possible emissivity values for every pixel of the reconstructed thermal image.

We have therefore characterized both $\varepsilon_{\text{glass}}$ and $\varepsilon_{\text{PBNP}}$ by the black-tape method. Based on the results, $\varepsilon_{\text{glass}} = 0.93$ and $\varepsilon_{\text{PBNP}} = 0.97$, we have subsequently adopted an average emissivity of 0.95 for the datasets collected on all the murine biopsies (note $\varepsilon_{\text{PBNP}}$ does not differ significantly from the emissivity of bare skin, values between 0.95 and 0.98 being reported in the literature for humans, mice and several mammals[10-12]). At the same time, we have exploited the range $\varepsilon_{\text{glass}}$-$\varepsilon_{\text{PBNP}}$ to quantify, at each pixel of the reconstructed images, how much the uncertainty on the emissivity value propagates to the uncertainty on temperature variations. Significantly, when we analyze the very same dataset of **Fig.3e** with space-independent emissivities = 0.93, 0.95 or 0.97, nearly identical maps of temperature variations are obtained: the uncertainty on the emissivity value in the range 0.93-0.97 does not hamper the thermal reconstruction of the nanoparticles distribution inside the tissue, and allows to estimate the local temperature increments with a maximum uncertainty of 0.1°C (**Supplementary Fig.9**). Based on these considerations, we can also conclude that the assumption of uniform emissivity does not affect sensibly the temperature values measured in **Fig.3** of this work.

5.2 Effect of emissivity or thermal diffusivity heterogeneities

The microfiche samples we have employed for proof-of-principle experiments in **Fig.2** are homogeneous in terms of emissivity. Similarly, the range of possible emissivity values in PBNP-treated murine skin sections is sufficiently small to translate into a negligible (0.1 °C) uncertainty in measured temperature increments. It is however worth discussing the applicability of the proposed approach in case of heterogeneous samples, the heterogeneity involving the emissivity and/or the sample thermal diffusivity.

Micro-absorbers embedded in patches of the sample with different thermal diffusivity, or characterized by different emissivity values, would be included in the reconstructed images. However, spatial heterogeneities of the sample thermal diffusivity D and emissivity ε would possibly impact on: (i) the accuracy in the measurement of $\Delta T_{\max}$ values, and (ii) the variability of the signal/noise ratio over different regions on the acquired thermal camera frames, that in turn regulates the peaks localization uncertainty and the achieved resolution.

Regarding point (i), the accuracy in the measurement of temperature increments from the Stefan-Boltzmann law is affected only by the emissivity heterogeneity, that would result in apparent point-by-point variations in the $\Delta T_{\max}$ values. Such an effect, that could only be corrected for with pixelwise prior knowledge of the emissivity value, affects any infrared thermal imaging technique. We expect instead no effect on the accuracy in the measurement of the temperature increments from the heterogeneities in thermal diffusivity: the presence of different D values would result in temperature peaks with physically meaningful different amplitudes (as remarked in the theoretical treatment of **Supplementary Note 1**), but these would be correctly revealed by the peak fitting procedure.

Regarding point (ii), since the localization accuracy decreases when the emitted power decreases (**Supplementary Fig.5e**), the sample heterogeneity (both in terms of thermal diffusivity and emissivity) might affect the spatial resolution of the image. If the sample is highly heterogenous, we expect to find patches in the sample where we can reconstruct the distribution of the absorbers with an accuracy higher than in other patches. However, from **Supplementary Fig.5e**, we see that at $\Delta T_{\max} \cong 1^{\circ}$C a 10% change in the measured temperature variation only implies a 7% change of the localization accuracy. Lowering to $\Delta T_{\max} \cong 0.5^{\circ}$C, the same 10% change in the measured temperature variation would produce a 9% variation in the position accuracy. We argue this variability in the localization precision, arising from variations of the diffusivity or emissivity from points to points of the sample, would smooth some details in the reconstructed images without affecting sensibly the overall morphological reconstruction of the sample.

5.3 Substrate effects

We finally discuss the limitations of the proposed super-resolution approach in terms of (i) sample semi-transparency in the far-infrared wavelength range, and (ii) thermal diffusivity of both the sample and a possible substrate.

The semi-transparency of the sample in the far-IR range would produce an undesired bias in the measured temperature variations. Part of the heat dissipated by the sample would heat the substrate up, and the substrate thermal emission would be collected by the bolometer through the semi-transparent sample. The correction to the measured temperature increment would then depend on the emissivity of the substrate (more photons than expected being collected, especially when the emissivity of the substrate is higher than the emissivity of the absorbing sample layer). However, this effect can be mitigated by a direct measure (for example, by the black-tape method) of the emissivity of the sample laying on the substrate. In fact, if the sample is semitransparent to the thermal radiation, we could actually measure an effective emissivity of the sample+substrate structure (the photon contributions of both the sample and the substrate would be simultaneously taken into account when determining the emissivity value). We do not expect instead an effect of the thermal diffusivity of the substrate on the accuracy with which we recover the peak position of the temperature distribution. The temperature distribution of the substrate imaged through the (partially transparent) sample layer would only produce an approximately Gaussian background below the sharper peak due to the sample absorption: it will not shift its peak position. We expect therefore that our peak center localization algorithm will work as well in the presence of a semi-transparent sample overlaid to a substrate with different thermal diffusivity.

Regarding the range of thermal diffusivities on which our algorithm can still provide us with a map of the absorbers, we remark that:

- The maximum temperature increase under laser illumination is approximately inversely proportional to the sample thermal diffusivity (**Supplementary Note 1** and **Supplementary Fig.1b**);
- The minimum signal/noise ratio for which we can fit a Gaussian peak with an uncertainty in the position of $\cong \frac{1}{3}$ of the nominal spatial resolution of the thermocamera is $\cong 2$ (**Supplementary Fig.5e**).

We take as a reference the situation reported in **Fig.2c**, that corresponds to a laser spot intensity of 0.6 kW/cm^2, an emissivity $\varepsilon = 0.8$ and a diffusivity D = 5·10^5 μm^2/s (measured with a modification of the laser flash method[4]). This situation corresponds to an average signal/noise ratio S/N $\cong$ 9. Since we can detect by means of the Gaussian peak fitting the presence of absorbers down to a S/N = 2 with a spatial resolution of about 100 μm, we can say that the maximum thermal diffusivity of the sample for which we can recover a distribution map, at the excitation intensity used here, is $D_{\mathrm{T,max}} \cong\ 23.5 \cdot 10^5$ μm^2/s. This value can be increased by raising the laser intensity, provided that there is no photo-damage of the sample. With the samples used here, the intensity can be easily raised by four times without damaging the sample (**Fig.2g**), increasing the maximum value of the diffusivity of the same ratio up to $D_{\mathrm{T,max}} \cong 9 \cdot 10^6$ μm^2/s. On the lower limit, the minimum thermal diffusivity of the sample on which we can work is set by the relaxation time of the temperature jump compared to the line scanning rate: by increasing the time interval in between consecutive illumination events, we can tackle low sample diffusivities with the only drawback of the increased total data acquisition time. We conclude therefore our method can be applied to any thermal diffusivity value in the broad range 10^4-10^7μm^2/s, covering practically any material from PVC to steel.

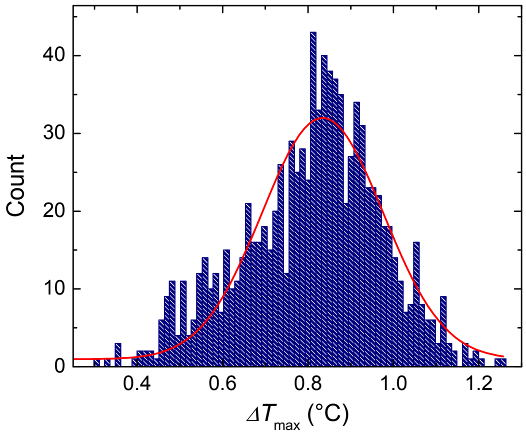

Supplementary Figure 6: histogram of ΔT_{max} values in the super-resolution image of Figure 2c. Histogram of ΔT_{max} values over all the pixels of **Fig.2c**. The standard deviation (0.14±0.01) °C of the Gaussian fit is close to the thermal camera sensitivity σ_T = 0.1°C as stated by the producer (FLIR Inc., USA); the distribution of detected temperature variations in **Fig.2c** is therefore compatible with the high sample homogeneity, in terms of ink distribution, shown by the transmitted-light image (**Fig.2a**) of the sample.

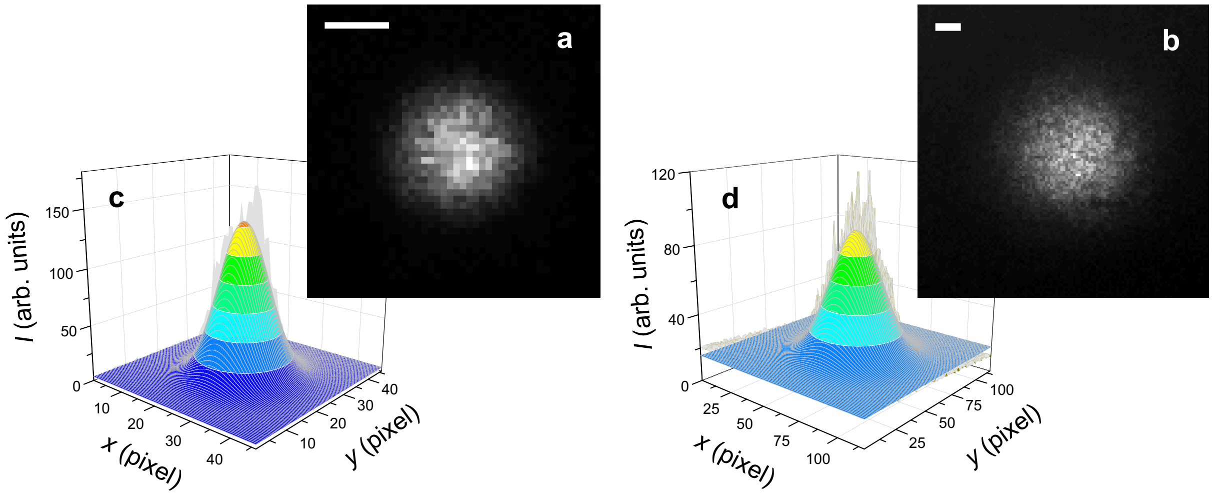

Supplementary Figure 7: measurement of the laser spot size. (a),(b) Image of the He-Ne laser spot, incident on a black screen, acquired by a CMOS camera (IDS Imaging Development Systems, D) at a distance z = (43±1) cm beyond the z = 0 focal point, in the absence of beam-reducing unit (i.e., without lenses L1 and L2 in **Supplementary Fig.3a**) in (a), and in the presence of the beam-reducing unit (f_1=3 cm and f_2=10 cm in **Supplementary Fig.3a**) in (b); scale bar = 3 mm. (c) Gaussian surface fit of the image in (a) to $I(\mathbf{x}) = Aexp(-2\mathbf{x}^2/\rho_z^2)$, with best-fit 1/e² radius $\rho_{z=43cm}$=(3110±92) μm. The laser beam waist ρ_0 at the focal point z = 0 is derived from the recovered beam waist $\rho_{z=43cm}$ according to $\rho_z = \rho_0\sqrt{1+[\lambda z/(\pi\rho_0^2)]^2}$. Solving the quartic equation for ρ_0 leads to $\rho_0 = \frac{1}{\sqrt{2}}\sqrt{\rho_z^2 - \sqrt{\rho_z^4 - 4(\lambda z/\pi)^2}}$=(28±1) μm. (d) Gaussian surface fit of the image in (b) with best-fit 1/e² radius $\rho_{z=43cm}$ = (8091±346) μm, leading to ρ_0 = (10.7±0.5) μm for the beam-reducer configuration. The result obtained in the absence of the beam reducer has been further confirmed (data not shown) by a separate measurement of the laser spot size based on the variance of the spatial auto-correlation of the speckle field produced by a coarse tape surface positioned at the focal point of the laser beam and imaged by the same IDS camera at distance z = (43±1) cm.

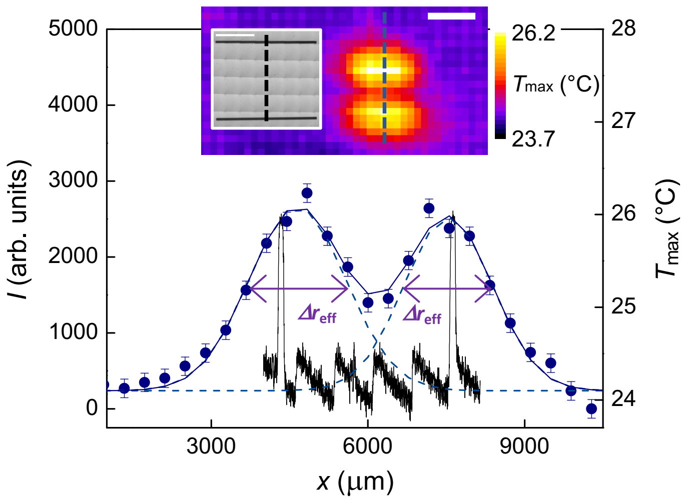

Supplementary Figure 8: thermal-camera resolution. Solid black line: intensity profile along the black dashed line in the transmission microscopy image (left inset; scale bar 1.66 mm) of two parallel ink stripes printed as microfiche (90 μm stripes width, 3.3 mm stripes distance). The sawtooth trend is due to the tile-scan procedure exploited to image the extended sample by transmitted-light microscopy (λ_{exc} = 633 nm). Blue circles: temperature profile along the blue dashed line in the thermographic image (right inset, 2.7 mm scale bar) obtained as a signal maximum projection over the time axis of the image stack acquired during fast bidirectional laser raster-scanning of the ink sample (P = 18 mW, pixel dwell time 3 ms, λ_{exc} = 633 nm). The fit of the temperature profile to a two-component Gaussian function provides an average full width at 60% amplitude Δr_{eff} = (1850±80) μm. Δr_{eff} depends here on the excitation laser power at fixed thermal camera sensitivity σ_T (since the image is derived as a signal maximum projection over the whole time sequence of images, the observed width Δr_{eff} corresponds to the maximum width reached by the temperature profile before falling below the σ_T level; at higher laser power, the temperature drops below σ_T at a later time point, when heat diffusion produces a broader temperature profile).

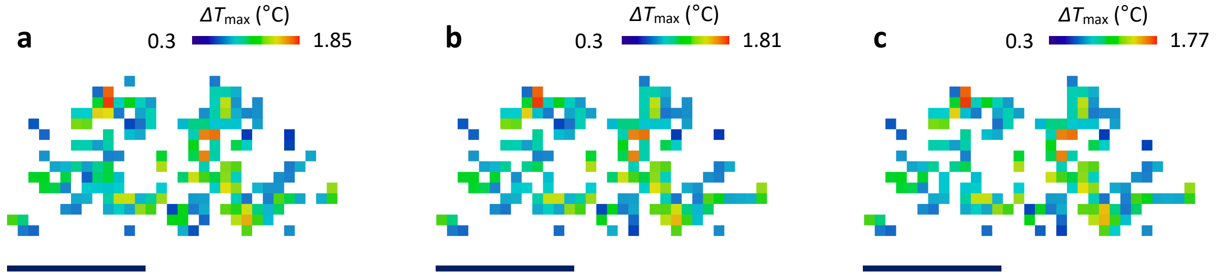

Supplementary Figure 9: effect of emissivity heterogeneity on treated skin biopsies. (a)-(c) Super-resolution photo-thermal images of the same data-set reported in **Fig.3e** upon analysis with constant emissivity of 0.93 (a), 0.95 (b) and 0.97 (c). Scale bar = 1 mm.

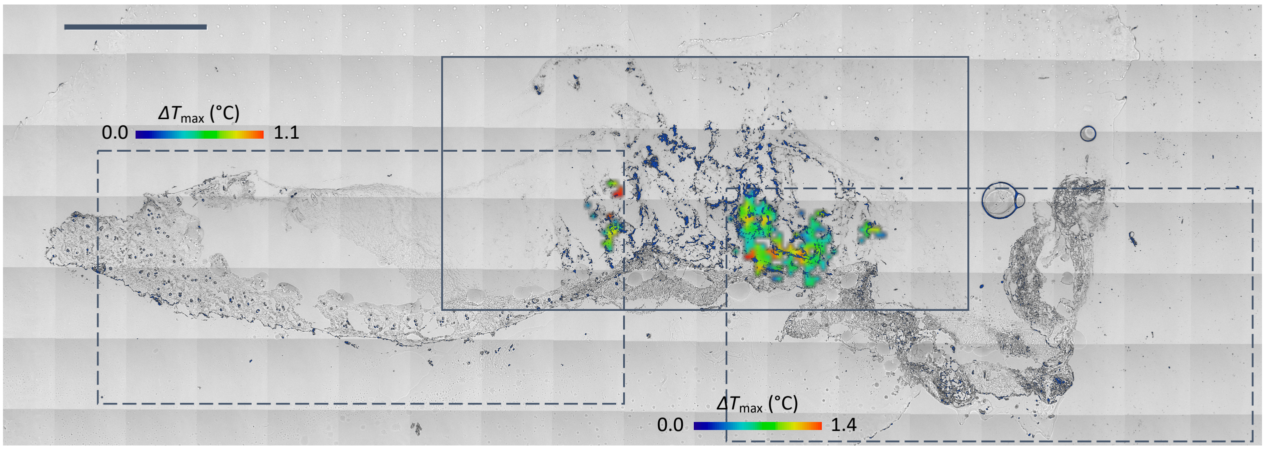

Supplementary Figure 10: super-resolution imaging of nanoparticles-treated murine biopsies. Transmitted-light tile-scan image at 633 nm of the explanted murine skin biopsy treated with 30-nm PB nanocubes exploited for the experiments reported in **Fig.3** (main text); nanocubes are highlighted in navy by an upper threshold on the intensity counts. In the dashed ROIs the transmitted-light image is overlaid to the super-resolution photo-thermal images acquired with N_xxN_y = 100x48, δx = 37.6 μm, Δx = 50, Δy = 1, τ_{on} = 1 s, P = 15 mW, laser beam 1/e^2 diameter 22±1 μm (ΔT_{min} = 0.3°C). We remark that no endogenous temperature variation is detected outside the area of nanoparticles injection. Scale bar = 1 mm. The super-resolution image of ROI 1 (boxed region) is reported in **Fig.3**.

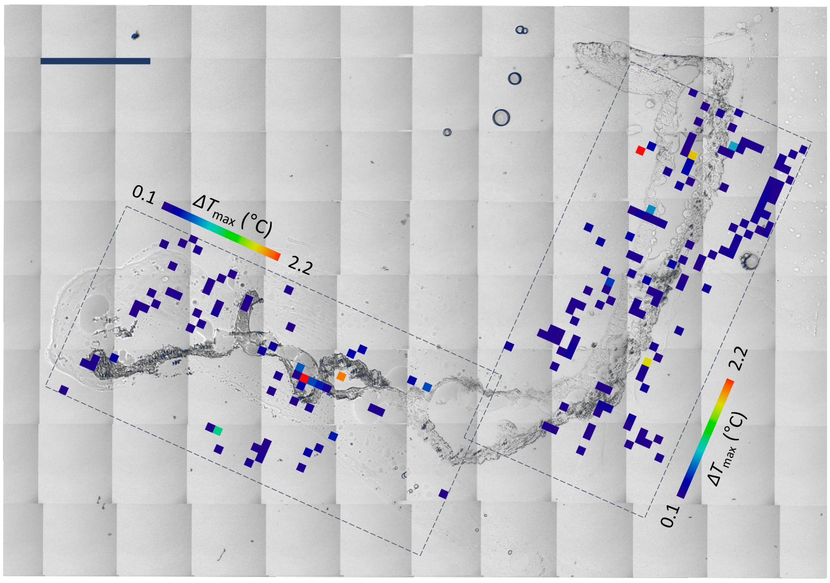

Supplementary Figure 11: super-resolution imaging of untreated murine biopsies. Transmitted-light tile-scan image at 633 nm of the untreated explanted murine skin biopsy exploited for the experiments reported in **Fig.3** (main text). In the ROIs denoted as 2 and 3 in **Fig.3** (dashed areas) the transmitted-light image is overlaid to the super-resolution photo-thermal images acquired with parameters N_xxN_y = 50x24, δx = 75.3 μm, Δx = 25, Δy = 2, τ_{on} = 1 s, P = 15 mW, laser beam 1/e^2 diameter 22±1 μm (ΔT_{min} = 0.1°C). Scale bar = 1 mm.

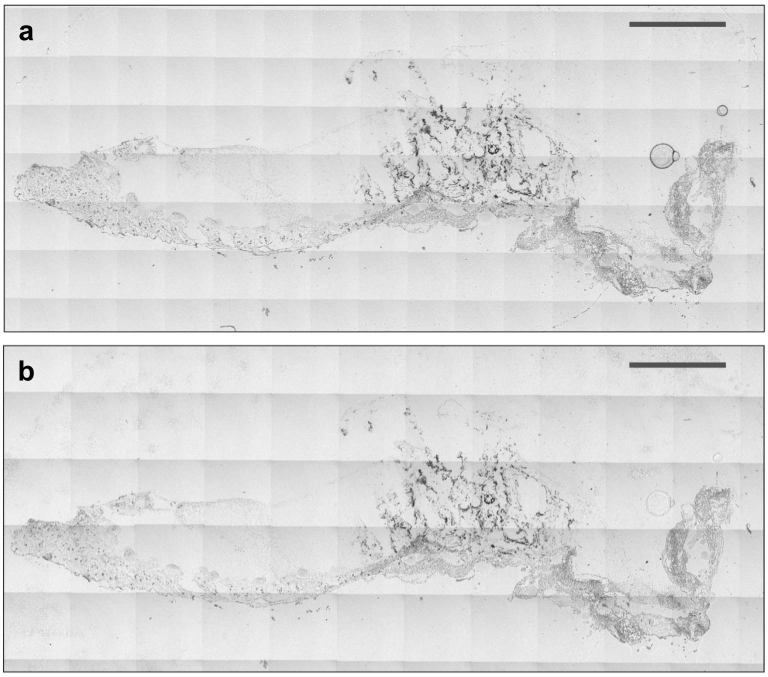

Supplementary Figure 12: absence of photo-damage on murine skin biopsies. (a),(b) Transmitted-light tile-scan images at λ_{exc} = 633 nm of the explanted murine skin biopsy treated with Prussian blue 30-nm nanocubes exploited for super-resolution photo-thermal imaging in **Fig.3**. Images have been collected under identical conditions before (a) and right after (b) the 90-min data acquisition of **Fig.3** to demonstrate the absence of sample photo-damage induced by modulated laser light illumination at 15 mW power. Scale bars = 1 mm.

Supplementary Note 6: Scan path calibration

As schematically depicted in **Supplementary Fig.3**, two galvanometric mirrors are employed to scan the laser beam on the sample plane along a conventional raster pattern, with grid size N_xxN_y and pixel size δx. Mirrors are operated by means of a Python code, which drives in turn an Arduino Uno microcontroller board based on the user-defined values for N_x, N_y, for the laser pixel dwell time and for the voltage V to be supplied to the mirrors.

The voltage V assigns the scan path length according to a linear relation with proportionality constant c. For simplicity, a single voltage value V is provided to the code to define the path length L_x along the horizontal x-axis according to $L_x = cV$, whereas the y-axis path length L_y is implicitly defined through the number of pixels N_y and computed as $L_y = L_x N_y / N_x$. The proportionality constant c depends on the lenses of the adopted scanning system, so that the conversion of the voltage value into the raster-pattern length on the sample plane has to be experimentally calibrated. The corresponding pixel size $\delta x = L_x / N_x = cV / N_x$ of the scanning grid can then be exploited to assign the pixel size in the reconstruction of the final super-resolution image.

The procedure we have developed to calibrate the scan path by means of the thermal camera takes advantage of a uniformly absorbing sample of known size, sharp edges and preferably square or rectangular shape. For the present work, we have exploited an ink square, produced via microfiche printing, with sides $\ell_x = \ell_y \equiv \ell$ = 0.4 cm (**Supplementary Fig.13a**). The scan path should completely cover the sample, as schematically depicted in **Supplementary Fig.13b**: to this aim, mirrors are supplied by a given voltage V (3V, in the present case), and complete coverage of the sample by the resulting raster scan path is visually checked. If this condition is satisfied (L_x, $L_y > \ell$), for each scan line lying within the ink square the laser beam scans N_1 pixels before actually illuminating the sample, then it scans N_2 pixels inside the sample square, and it finally scans N_3 pixels outside the ink (**Supplementary Fig.13b**). Recalling that the known user-defined value N_x must satisfy (N_1+N_2+N_3) = N_x, the

measurement of N_1 and N_3 should allow recovering N_2; then, N_2 can be exploited to quantify the scan pixel size δx according to $\delta x = \ell/N_2$, as well as the scan path length according to $L_\mathrm{x} = N_\mathrm{x}\delta x = N_\mathrm{x}\ell/N_2$. The resulting calibration constant $c = N_\mathrm{x}\ell/(N_2 V)$ provides the conversion factor to compute L_x and δx for any voltage value subsequently provided to the mirrors. Even though in principle a single line scan is sufficient to recover N_1, N_2, N_3 and δx, for statistical convenience the same procedure is repeated for the all the scanned lines contained within the raster pattern.
The measurement of N_1 and N_3 can be performed by exploiting the time-series of images acquired by the thermal camera during the laser scanning. Differently from the mirrors operation during super-resolution photo-thermal imaging, the raster pattern is scanned by the laser beam in bi-directional mode, with constant pixel dwell time (here, 200 ms) and no modulation of the laser illumination (i.e., the shutter is permanently open). Provided the scan pixel dwell time τ_d is known, at the j-th line of the raster pattern, N_1 can be determined as

$$N_1 = \frac{t'_j - t_\mathrm{start}}{\tau_\mathrm{d}} - 2(j-1)N_\mathrm{x} \qquad (19)$$

t_start is the time when the laser starts scanning at position (1,1), while t'_j is the time point when the laser hits the first pixel of the j-th line that lies within the absorbing region of the sample. Such a pixel will be hereafter referred to as pixel $p'_j=(i', j)$ (**Supplementary Fig.13b**). With these definitions, N_1 is derived as the difference between the number of pixels scanned during the time it takes to the laser beam to reach pixel p'_j at the j-th line, minus the total number of pixels ($2(j-1)N_\mathrm{x}$) scanned bi-directionally in the preceding j lines.
Supplementary Equation 19 requires the estimates of t_start and t'_j. The thermal camera is not synchronized with the scan system, and starts acquiring frames at time $t < t_\mathrm{start}$; since no thermal increase is detected by the thermo-camera at time t_start (the beam does not illuminate an absorbing region of the sample), a stepper motor has to be connected to the same Arduino board driving the galvanometric mirrors and inserted into the thermo-camera field of view. The stepper motor turns 180° when the scan start is triggered and, by appearing in the thermal images, it allows identifying t_start with an uncertainty equal to the inverse of the thermo-camera frame rate f_rate. Automatic and reliable determination of t'_j from the thermal camera image sequence is less straightforward, since the exact spatial location of the scan pixel of interest (pixel p'_j, lying at the edge of the ink square) within the thermo-camera pixel grid is not even known. In order to identify the image column that contains the ink edge and p'_j, we found it convenient to isolate a Region Of Interest (ROI, R_xxR_y) in the images to encompass the entire scanned region (it can be easily identified starting from the maximum projection of the acquired image stack, shown in **Supplementary Fig.13c**), and to average the thermal signal column-wise across the ROI. The averaged signals can either be plotted versus time as $T_I(t)$ profiles for each I-th column (**Supplementary Fig.13d,e**; I=1.. R_x), or reported as $T_I(t)/\,T_{I+1}(t)$ profiles (**Supplementary Fig.13f,g**). In principle, if we call I' the x-axis coordinate of the pixel of the thermal images that contains scan pixel p'_j (**Supplementary Fig.13c**), I' can be identified by the first column index, moving from left to right, where the corresponding intensity profile $T_{I'}(t)$ shows distinct temperature peaks. However, identification of such a profile is complicated by the fact that when the laser beam hits the ink border in column I', heat diffusion effects and the effective (1850±50 μm) thermo-camera resolution lead to the detection of an increase in the thermal signal also in a few columns with index $I < I'$ (**Supplementary Fig.13c**). We found that correct identification of I' is easier when $T_I(t)/\,T_{I+1}(t)$ profiles are considered. These profiles have constant value 1 for all the columns where no significant thermal increase is detected; by contrast, any time the laser beam induces a different temperature variation in nearby columns of index I and $I+1$, profile $T_I(t)/\,T_{I+1}(t)$ shows a peak: the position along the time axis of each odd peak defines the time point when the laser shifts from column I to column $I+1$ (even peaks refer to the transition from column $I+1$ to column I during backward scanning, and are neglected for the determination of N_1). I' can be therefore determined as the first column index, moving from the left to the right, where the corresponding intensity profile $T_{I'}(t)/\,T_{I'+1}(t)$ shows clearly distinct peaks (**Supplementary Fig.13g**).
Once profile $T_{I'}(t)/\,T_{I'+1}(t)$ has been identified (**Supplementary Fig.13g,h**), odd peaks get counted and the frame index along the time axis where peaks occur is stored to provide the sequence of values t'_j at

increasing j. Together with the estimated time t_{start}, t'_j values can be substituted into Supplementary Equation 19 to retrieve N_1 as a function of j (**Supplementary Fig.13i**). For the specific data-set reported in **Supplementary Fig.13**, the average N_1 is N_1 = (30±1).
The measurement of N_3 proceeds in exactly the same way. If we call t''_j the time point when the laser first hits the absorbing region of the sample while scanning backwards (from the right to the left) along the j-th line, N_3 can be derived as

$$N_3 = \frac{t''_j - t_{\text{start}}}{\tau_{\text{d}}} - [2(j-1)+1]N_{\text{x}} \qquad (20)$$

As in the case of t'_j, t''_j is measured by inspecting $T_I(t)/\,T_{I-1}(t)$ profiles (I=N_{x}...1) and by identifying the I'' column containing the ink square edge. Starting from the corresponding $T_{I''}(t)/\,T_{I''-1}(t)$ profile (**Supplementary Fig.13h**), the sequence of t_j'' values as a function of j is retrieved by the position along the time axis of the even peaks. For the data-set of **Supplementary Fig.13**, N_3 = (37±1) (**Supplementary Fig.13i**). N_1 and N_3 allow recovering N_2 = (32±1), leading to δx = ℓ/N_2 = 124±4 μm (**Supplementary Fig.13i**) and c = $N_{\text{x}}\ell/(N_2 V)$ = 4170±130 μm/V.

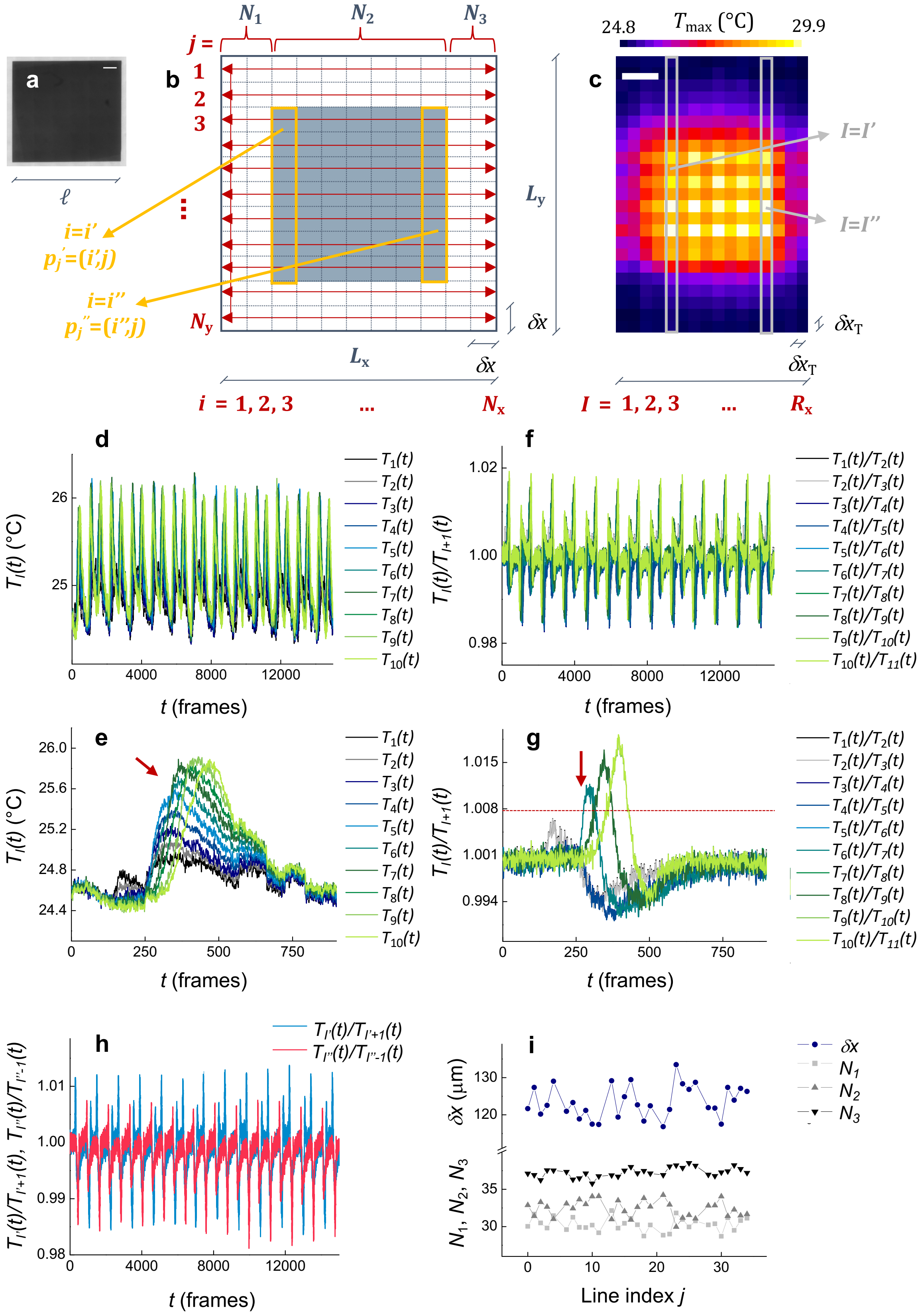

Supplementary Figure 13: scan path calibration. (a) Transmitted-light image at 633 nm of a uniform microfiche square. Scale bar=500 μm. (b) Raster-pattern with grid format N_xxN_y, pixel size δx and scan path lengths L_x and L_y, scanned bi-directionally along the red arrows (here V=3V, N_x=N_y=100,

τ_d=200 ms). The scan path covers the sample, shown in grey. Orange columns identify the scan pixels along the vertical edges of the square: pixels $p_j'=(i', j)$ and $p_j''=(i'', j)$ are those where the laser beam hits the absorbing region during forward and backward scanning, respectively. We assume the sample is aligned to the scan grid, so that the i' and i'' coordinates of pixels p_j' and p_j'' are the same for any j. (c) ROI extracted from the temporal maximum projection of the thermo-camera image sequence acquired during bidirectional scanning across the ink square in (a); the ROI size R_xxR_y includes the entire scanned area. Scale bar=1230 µm, thermal-camera pixel size on the sample plane δx_T=410 µm. Grey bars highlight the image columns superimposed to the vertical ink edges, identified starting from the profiles in (d)-(h) as described in the text. (d) Profiles $T_I(t)$ (for I=1…10) computed over the ROI of panel (c): at each time point, $T_I(t)$ is computed as a spatial average of the signal over the ROI I-th column. Out of the 42060 acquired frames, the first 15000 time points are shown for the sake of display. (e) Same profiles of (d), magnified on the 0-900 frames time scale to highlight the temperature variations detected during the first forward line scan. (f) Temporal profiles $T_I(t)/T_{I+1}(t)$ (for I=1…10) over the ROI of (c) on the 1-15000 frames time scale. (g) Same of (f), magnified on the 0-900 frames time scale of the first forward line scan. The red arrow marks the profile $T_{I'}(t)/T_{I'+1}(t)$, identified as the first profile where peaks appear over a predefined threshold (dotted line). The corresponding profile $T_{I'}(t)$ is indicated in (e) by the arrow. (h) Profiles $T_{I'}(t)/T_{I'+1}(t)$ and $T_{I''}(t)/T_{I''-1}(t)$ computed in the ROI columns of index I' and I''. (i) Recovered values for N_1, N_2, N_3 and δx.

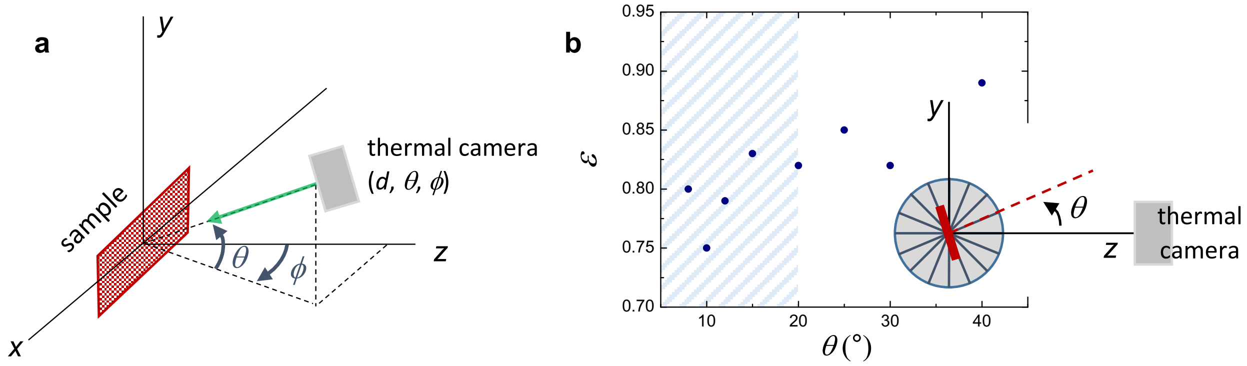

Supplementary Figure 14: emissivity angular dependence for microfiche samples. (a) Sketch of the thermal camera orientation with respect to the sample. The thermal camera, at distance d, points to the sample along the green arrow; ϕ defines the tilt in the horizontal xz-plane, whereas θ defines the vertical tilt in the yz-plane ($\phi = \theta = 0°$ corresponds to normal sample observation). (b) Emissivity of the microfiche samples (ink printed areas) as a function of the observation angle θ at fixed $\phi = 0°$. All the super-resolution imaging experiments of the present work have been performed with $\theta < 20°$ where the average emissivity equals $\varepsilon = 0.80 \pm 0.03$ (mean ± standard dev.). Inset: schematic of the setup for the measurement of the emissivity angular dependence. The microfiche sample (red) is attached to an angular scale, and half of the sample front surface is covered with black matte tape of known emissivity (ε_{tape}=0.95 independently from the observation angle): under thermal equilibrium, the object emissivity can be found by varying ε in the camera software until the thermal image provides the same temperature for the sample and the tape. The procedure is repeated at increasing θ values by rotating the object to retrieve the emissivity dependence on the angle.

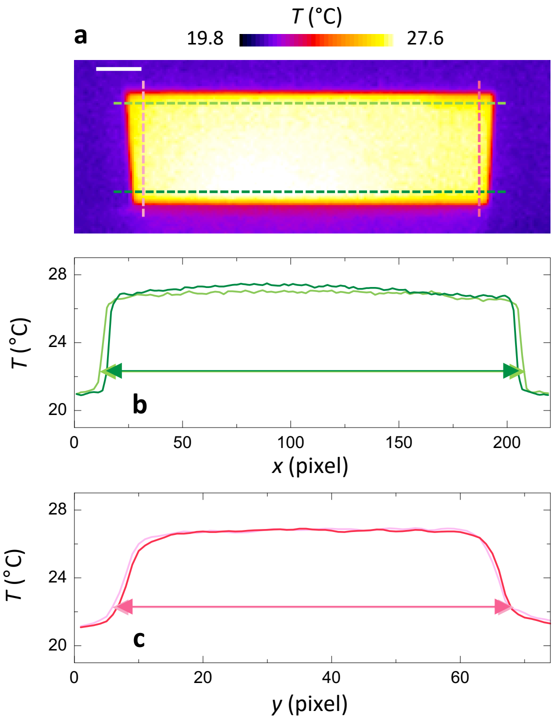

Supplementary Figure 15: effect of the thermal camera viewing angle. (a) Thermal image of a heated glass slide (7.65cm x 2.5cm in size, emissivity ε = 0.93) employed to quantify the effect of the thermal camera viewing angle with respect to the sample and derive the thermal camera pixel size on the sample plane (scale bar, 1 cm). The image has been acquired with a 20° thermal camera tilt along the vertical direction, and no tilt in the horizontal direction (as depicted in **Supplementary Fig.14**, with ϕ = 0° and θ = 20°). This condition corresponds to the highest possible deviation from normal sample observation in the imaging experiments of the present work, that have been typically performed with ϕ = 0° and θ = 10°-20°. (b) Temperature profiles extracted from the color-coded dotted horizontal lines in panel (a). The profiles length, as represented by the arrows, varies from 197 to 191 pixels, with only a 6-pixels (3%) foreshortening effect due to perspective view. A 395±8 μm horizontal thermal camera pixel size δx_T on the sample plane is obtained from the profiles and the known 7.65-cm object length. (c) Temperature profiles extracted from the color-coded dotted vertical lines in panel (a), with a 61±1 pixels length. A 404±4 μm vertical thermal camera pixel size δy_T on the sample plane is obtained from the two profiles and the known 2.5-cm object width. The compatibility of the δx_T and δy_T pixel sizes allows assuming a square 400±6 pixel size on the sample plane (for simplicity, δx_T will denote the square-pixel size everywhere in the text).

Supplementary References

1. Wollmer, M. & Moellmann, K. P. *Infrared Thermal Imaging. Fundamentals, Research and Applications, Ch. 2* (Wiley-Vch, Weinheim, 2010).
2. Mathematica, Wolfram Research, Inc., Champaign, IL, USA (2018).
3. Peah-May, N. W., Mendioroz, A., & Salazar, A. Simultaneous measurement of the in-plane and in-depth thermal diffusivity of solids using pulsed infrared thermography with focused illumination. *NDT&E International.* **77**, 28-34 (2016).
4. Cernuschi, F., Russo, A., Lorenzoni, L. & Figari, A. In-plane thermal diffusivity evaluation by infrared thermography. *Rev. Sci. Instrum.* **72**, 10, (2001).
5. Salazar, A. et al. extending the flash method to measure the thermal diffusivity of semitransparent solids. *Meas. Sci. Technol.* **25**, 035604 (2014).

6. Tian, W., Wang, C., Wu, M. & Wang, T. The multivariate extended skew normal distribution and its quadratic forms. In *Causal Inference in Econometrics* (Springer International Publishing Switzerland, 2006).
7. Azzalini, A. & Dalla, A. The multivariate skew-normal distribution. *Biometrika.* **83**, 4, 715-726 (1996).
8. User's manual FLIR E40 Series, FLIR Systems Inc.
9. Ball M. et al. Factors affecting the accuracy of thermal imaging cameras in volcanology, *J. Geophys. Res*. **111**, B11203 (2006).
10. Lahiri, B.B., Bagavathiappan, S., Jayakumar, T. & Philip, J. Medical applications of infrared thermography: a review. *Infrared Phys. & Technol.,* **55**, 221-235 (2012).
11. Mortola, J.P., Thermographic analysis of body surface temperature of mammals, *Zool. Sci.,* **30**, 2, 118-124 (2013).
12. Polymeropoulos, E.T. et al. *The evolution of endothermy – from patterns to mechanisms,* Frontiers in Physiol. (2018).
